# COVID-19 unit in psychiatric hospital

**DOI:** 10.1192/j.eurpsy.2021.706

**Published:** 2021-08-13

**Authors:** I. Alonso Salas, C. González Navarro, O. Euba García

**Affiliations:** Psychiatry, Hospital de Zamudio, Zamudio, Spain

**Keywords:** Covid, Psychiatric hospital, integral care, psicosis

## Abstract

**Introduction:**

As coronavirus pandemic burst in Spain in March 2020, Zamudio Hospital -a monographic psychiatric institution- was urged to create an specific Covid unit. It was destined to patients with psychiatric admission criteria, who in addition oscillated between positive asymptomatics or with mild symptoms to suspect cases or close contacts.

**Objectives:**

To describe and analyse the characteristics of the unit and the patients who were admitted during the confinement period by Covid-19, between March 14th and June 21^st^ 2020.

**Methods:**

The patients’ data were collected retrospectively. These data included: age; sex; admission criteria; diagnosis at discharge; confirmed/ suspected/contact case; presence/absence of symptoms; length of hospital stay; number of doctor on call assessment.

**Results:**

An area within the hospital wards was reserved to COVID cases / suspected / contact patients requiring psychiatric care. The storing of material and PPE was held in the forementioned area, according to protocolary measures. 26 Patients (11 women and 15 men) were admitted to the unit. Mean age was 44 years old. Diagnosis at discharge were mainly Schizophrenia (31%), Schizoaffective disorder (23%), other psicosis (11,5%) and Bipolar disorder (8%). The mean hospital stay was 5 days. There were a total of 7 confirmed positive cases, all with asyptomatic-mild course.

**Conclusions:**

The establishment of this unit has ensured a proper psychiatric care and a strict control of Covid-19 transmission within patients and staff members.

